# P41 Combating periodontal disease: chitosan-FK13a1 microparticles as a promising antibiotic alternative

**DOI:** 10.1093/jacamr/dlaf230.048

**Published:** 2025-12-04

**Authors:** Samaneh Keshavarz, Josefine Hirschfeld, Melissa Grant

**Affiliations:** Dentistry, School of Health Sciences, College of Medicine and Health, University of Birmingham, 5 Mill Pool Way, Edgbaston, Birmingham, B5 7EG, UK; Dentistry, School of Health Sciences, College of Medicine and Health, University of Birmingham, 5 Mill Pool Way, Edgbaston, Birmingham, B5 7EG, UK; Dentistry, School of Health Sciences, College of Medicine and Health, University of Birmingham, 5 Mill Pool Way, Edgbaston, Birmingham, B5 7EG, UK

## Abstract

**Background:**

Due to antibiotic resistance in the present era the treatment of bacterial infections has become difficult. In dentistry, irregular administration of antibiotics has complicated the treatment of dental and periodontal infections. Thus, the prescription of antibiotics should be reduced either by finding acceptable alternatives or by effective adjunctive therapies aimed at reducing the dose of antibiotics. Antimicrobial peptides (AMPs) have shown broad-spectrum antimicrobial activity, including effectiveness against periodontal pathogens. LL37 is one of the natural AMPs in the human body, and one of its derivatives, FK13a1, was tested in the present study. One of the problems with working with AMPs is that they lose their effectiveness in aqueous solutions. One strategy for protecting these peptides is to encapsulate them in biomaterials such as chitosan, which, by forming microparticles, increases their stability, possibly allowing for controlled release of the peptide.

**Methods:**

In this study, chitosan and tripolyphosphate are used to produce these microparticles through cross-linking to enable AMPs to be used in oral products, including mouthwashes. Peptide release and their efficacy in inhibiting periodontal bacteria are compared with the efficacy of non-encapsulated peptide.

**Conclusions:**

The aim of this research is to employ a suitable method to stabilize AMPs and thus facilitate their clinical application in the fight against periodontal diseases, especially in patients whose periodontal tissue repair is impaired, such as diabetic patients or smokers.

